# Correlation between therapy and lipid profile of leprosy patients: is there a higher risk for developing cardiovascular diseases after treatment?

**DOI:** 10.1186/s40249-017-0295-1

**Published:** 2017-05-01

**Authors:** Rosalba V. G. Silva, Rafael S. de Araújo, Tinara L. S. Aarão, Paulo Diovanne da Silva Costa, Jorge R. Sousa, Juarez A. S. Quaresma

**Affiliations:** 1 Bettina Ferro de Souza Hospital, Federal do Para University, Belém, Pará Brazil; 2 Center of Health and Biological Science, Pará State University, Belém, Pará Brazil; 3 Tropical Medicine Center, Federal do Para University, Av. Generalissimo Deodoro 92, Umarizal, Belém, Pará 66055-190 Brazil

**Keywords:** Leprosy, Post-leprosy treatment, Nutritional status, Total cholesterol, Triglycerides, Cardiovascular disease, Brazil

## Abstract

**Background:**

The impact of leprosy reduces health-related quality of life of affected patients, interfering with different factors such as nutrition. This study investigated the lipid profile, nutritional status, and risk for cardiovascular disease (CVD) in patients who underwent leprosy treatment in Brazil.

**Methods:**

Eighty-four adult patients of both genders ranging in age from 20 to 60 years and diagnosed with paucibacillary (PB) or multibacillary (MB) leprosy were selected after undergoing multidrug treatment. The following data were collected: sociodemographic and clinical data; food intake; anthropometric measures (weight, height, and waist circumference); and lipid profile components (total cholesterol, high-density lipoprotein cholesterol [HDL-c], low-density lipoprotein cholesterol [LDL-c], and triglycerides).

**Results:**

Among the study population, there was a predominance of males (65.48%) aged 50 to 60 years, patients with an income of 248–496 American dollars (63.10%), patients who completed elementary school (65.48%), inactive patients (76.19%), non-smokers (46.43%), and non-drinking patients (69.05%). The levels (mean ± standard deviation) of total cholesterol were 193.8 ± 29.4 mg/dL in the PB form and 203.5 ± 41.7 mg/dL in the MB form. The mean LDL-c was 116.9 ± 22.7 mg/dL in PB patients and 121 ± 31.3 mg/dL in MB patients. Mean triglyceride levels were 123.4 ± 45.2 mg/dL in the PB form and 147.4 ± 88.9 mg/dL in the MB form. The evaluation of nutritional status showed that 41.67% of the patients were eutrophic, while 55.96% had excess weight. Food intake was significantly associated with HDL-c in male patients (*P* = 0.0264) and with triglycerides in patients above the ideal weight (*P* = 0.0049).

**Conclusions:**

The risk of acquiring CVDs was observed to be high due to patients’ excess weight and increased waist circumference. This study will guide clinicians in the adequate treatment of patients with leprosy in order to avoid adverse cardiovascular events.

**Electronic supplementary material:**

The online version of this article (doi:10.1186/s40249-017-0295-1) contains supplementary material, which is available to authorized users.

## Multilingual abstracts

Please see Additional file 1 for translations of the abstract into six official working languages of the United Nations.

## Background

Leprosy is a slowly progressing chronic, infectious and contagious, granulomatous disease caused by *Mycobacterium leprae*. The disease is endemic in Brazil and represents a serious public health problem in several countries [1]. According to the World Health Organization (WHO), 16 countries notified 1 000 or more cases in 2009. The highest rate of detection was found in Asia, with 9.39 cases per 100 000 inhabitants, followed by the Americas with 4.58 cases per 100 000 inhabitants [2].

In the Americas, among the 40 474 new cases, 93% were notified in Brazil until 2008, which continues to be the country with the second largest number of new cases in the world [3]. Pará state records one of the highest rates of new leprosy cases per year, with 45.48 cases per 100 000 inhabitants in 2008 [4].

An assessment of the population’s nutritional status is necessary to minimize nutritional risks and to promote a healthier lifestyle by identifying existing nutritional problems and developing appropriate interventions for patients [5]. Striuk et al. [6] emphasized the importance of combining anthropometry and lipid profile evaluation to complement nutritional assessments. According to Gupta et al. [7], the study of lipid metabolism in leprosy is important since lipid plays a central role in the pathology of the disease, and cholesterol dynamics in macrophages may influence the occurrence of cardiovascular diseases. Kumar et al. [8] suggested that lipids might also play an important role as an etiological agent of the vascular abnormalities observed in leprosy.

Several studies report the participation of lipoproteins in the genesis of cardiovascular lesions. The association between lipids and atherosclerosis has dominated the thinking of researchers until the beginning of the 1970s. At the end of the 20^th^ century, greater attention started to be paid to total cholesterol and high-density lipoprotein cholesterol (HDL-c) due to their important associations with the prevention or development of cardiovascular diseases (CVDs) [9].

Globally, as recent and more complete data about the lipid profile and nutritional status of patients with leprosy are lacking and considering the previous observation of lipid metabolism alterations in the different clinical forms of leprosy, a clinical nutrition study was conducted to describe and correlate the lipid profile, nutritional status, and risk for CVD in patients who underwent leprosy treatment.

## Methods

### Study subjects

84 adult patients of both genders with leprosy were selected for the study, between the years 2010 and 2012. The patients were registered and seen at the outpatient clinic of the Dr. Demetrio Medrado Specialized Referral Unit, State Department of Public Health, Belém, Pará, Brazil. 16 patients had the paucibacillary (PB) form and 68 had the multibacillary (MB) form.

### Inclusion and exclusion criteria

Patients of both genders ranging in age from 20 to 60 years who met the following inclusion criteria were included in the study: completion of the multidrug therapy regimen for leprosy at least two years and no more than five years ago; regular attendance to the specialized referral health unit (SRHU) for follow-up and physical rehabilitation with the multidisciplinary team; availability for sample and data collection; and voluntary agreement to sign the informed consent form after receiving detailed information about the study.

Criteria for exclusion were: patients not diagnosed with leprosy who had not been followed up regularly in the SRHU; age outside the range established for the study; incomplete data or lack of sociodemographic data or any other information that could not be obtained, especially data regarding blood collection and analysis; presence of diabetes mellitus; infection with human immunodeficiency virus; presence of a reactional state and recurrence of leprosy; diagnosis of dyslipidemias prior to the diagnosis of leprosy; and/or use of corticosteroids.

### Anthropometric parameters

Body weight and height were measured for the calculation of the body mass index (BMI). The BMI was determined by dividing the patient’s weight (kg) by his/her height squared (m^2^). The nutritional status of patients was classified according to the criteria of the WHO [10]. Body weight was measured with an anthropometric scale (capacity of 150 kg) to the nearest 100 g, according to the method of Ogawa et al. [11]. Height was measured with a stadiometer (range of 2.10 m) to the nearest 0.5 cm, as described by Chung [12].

Waist circumference (WC) was measured with a flexible inelastic measuring tape (length of 150 cm), calibrated in centimetres, at the midpoint between the last rib and upper border of the iliac crest [13]. The cut-off values recommended by the National Cholesterol Education Program-Adult Treatment Panel III were adopted to determine risk of comorbidities [14, 15].

### Food intake

A food frequency questionnaire was conducted to evaluate the food intake of the patients and included a list of foods divided into groups.

The method used for analysis of the food profile was adapted from Sichieri [14]. The rate of food consumption was calculated by transforming the reported frequencies into fractions of daily frequency. The following cut-offs were applied to classify the food intake level: < 0.33: low food intake; between ≥ 0.33 and < 0.66: intermediate food intake, and ≥ 0.66: high food intake.

### Sample collection and laboratory analysis

Disposable material was used for blood collection, which was disposed off according to laboratory safety standards. The material collected was used exclusively for the purpose of this study.

The patients were asked to fast for 12 h before blood collection. Venous blood samples (10 mL) were collected into vacuum tubes without anticoagulant to separate the serum. The collected material was identified with the patient’s data, stored, and sent to the Laboratory of Clinical Analysis of the Tropical Medicine Center/Federal do Para University for biochemical tests.

The samples were centrifuged at 3 500 rpm for 10 min to separate the serum, which was used to determine the lipid and lipoprotein concentrations. Triglycerides, total cholesterol, and HDL-c were measured biochemically using a spectrophotometer (semi-automatic TP Analyzer Plus®, Thermoplate, USA) applying a colorimetric enzymatic method, according to the recommendations of the manufacturers of method (Katal, Belo Horizonte, MG, Brazil; Doles, Goiânia, GO, Brazil). Low-density lipoprotein cholesterol (LDL-c) was calculated using the Friedewald equation, which is valid only when triglyceride levels are below 400 mg/dL. The reference values proposed by the 4^th^ Brazilian Guidelines on Dyslipidemias [15] were used to interpret the lipid profile.

### Statistical analysis

Descriptive and inferential methods were used to analyse the results. All variables were displayed using tabular and graphical methods to synthesize the data. The Shapiro-Wilk and D’Agostino-Pearson tests were applied to verify the normality of the quantitative variables. To analyse differences, the frequencies of the variables were compared using the following tests: chi-square test of independence Student’s *t*-test, and Mann-Whitney *U* test (for two independent samples). Pearson and Spearman’s rank correlation coefficients were calculated to determine the association between two quantitative variables. A level of significance of 0.05 (*P* = 0.05) was adopted and descriptive levels (*P*) below this value were considered to be significant. The GraphPad Prism 6 (USA) software was used for statistical analysis.

## Results

With respect to the sociodemographic characteristics of the population studied, there was a predominance of men (65.48%), patients aged 50–60 years (34.52%), patients who finished elementary school (65.48%) and had a monthly income of 248–496 American dollars (63.10%), non-drinking patients (69.05%), ex-smokers (46.43%), and inactive patients not doing any physical exercise (76.19%).

Table 1 shows the lipid profile (total cholesterol, HDL-c, LDL-c, and triglycerides) of patients according to the WHO operational classification. No significant difference (*P* > 0.05) was observed between groups for any of the variables.

**Table 1:** Lipid profile of patients with leprosy according to the WHO operational classification

Variable	Paucibacillary (*n* = 16)	Multibacillary (*n* = 68)	*P*
Total cholesterol (mg/dL)			
Mean ± SD	193.8 ± 29.4	203.5 ± 41.7	0.3809^a^
Median	192	200	
Range; CV (%)	143–250; 15.2%	104–291; 20.51%	
LDL-c (mg/dL)			
Mean ± SD	116.9 ± 22.7	121 ± 31.3	0.6222^a^
Median	110.5	116.5	
Range; CV (%)	84–163; 19.42%	48–210; 25.86%	
HDL-c (mg/dL), male			
Mean ± SD	51.7 ± 3.9	52.8 ± 8.6	0.5631^b^
Median	52	52	
Range; CV (%)	44–57; 7.47%	29–83; 16.24%	
HDL-c (mg/dL), female			
Mean ± SD	50.2 ± 8.1	53.5 ± 6.3	0.2832^a^
Median	48	55	
Range; CV (%)	40–62; 16.07%	38–64; 11.83%	
Triglycerides (mg/dL)			
Mean ± SD	123.4 ± 45.2	147.4 ± 88.9	0.5885^b^
Median	127.5	118.5	
Range; CV (%)	70–219; 36.65%	69–398; 60.34%	

*SD* standard deviation, *CV* coefficient of variation

^a^Student *t*-test

^b^Mann-Whitney *U* test

(*P* < 0.05)

The nutritional status of the population is shown in Table 2. Although a *p*-value of > 0.05 indicates a lack of statistical significance, it should be noted that more than half of the sample (47.56%) was either overweight or obese (39.29 and 16.67%, respectively).

**Table 2:** Nutritional status of leprosy patients

Classification of body mass index (kg/m^2^)	*n* = 84 (%)
Malnutrition (<18.5)	2 (2.38)
Eutrophy (≥ 18.5 < 25)	35 (41.67)
Overweight (≥ 25 ≤ 29.9)	33 (39.29)
Obesity (≥ 30)	14 (16.67)
*p*	0.3261

Chi-square test adherence with Yates correction. (*P* < 0.05)

On the basis of the reference values for the classification of the BMI, the mean total cholesterol concentration was 197 ± 40.8 mg/dL (median: 185 mg/dL) in the malnutrition and eutrophy categories. In overweight and obese patients, the mean total cholesterol was 205.3 ± 38.9 mg/dL (median: 203 mg/dL). The mean LDL-c concentration was 120.9 ± 31.7 mg/dL (median: 113 mg/dL) in malnourished and eutrophic patients, and 119.6 ± 28.5 mg/dL (median: 113 mg/dL) in overweight and obese patients. With respect to HDL-c in males, the mean concentration was 52.5 ± 6.3 mg/dL (median: 52 mg/dL) in the malnutrition and eutrophy categories, and 52.7 ± 9.1 mg/dL (median: 52 mg/dL) in overweight and obese patients. Mean HDL-c was 51.5 ± 8.5 mg/dL (median: 52 mg/dL) in malnourished and eutrophic female patients. In female overweight and obese patients, the mean HDL-c was 53.9 ± 4.8 mg/dL (median: 55.5 mg/dL). The statistical analysis (*P* > 0.05) showed no significant difference in total cholesterol, LDL-c, or HDL-c for either gender.

Mean triglyceride concentration was 120.4 ± 67.6 mg/dL (median: 89 mg/dL) in malnourished and eutrophic patients, and 160.4 ± 89.7 mg/dL (median: 132 mg/dL) in overweight and obese patients. There was a significant increase in triglycerides in the overweight and obesity categories (*P* = 0.0049).

Table 3 shows the increase of WC of the patients studied and a significant increase in CVD risk was observed in both genders (*P* < 0.0001).

**Table 3:** WCs of leprosy patients, according to gender

Sex	Normal	RCD	*P*
Male	*n* = 33	*n* = 22	
Mean ± SD	85.2 ± 6.1	100 ± 4.3	< 0.0001
Median	86	98.5	
Range; CV (%)	70.5–94; 7.16%	95–108; 4.33%	
Female	*n* = 7	*n* = 22	
Mean ± SD	77	92.8	< 0.0001
Median	74.7 ± 5.8	94.7 ± 11.7	
Range; CV (%)	63–80; 7.75%	81–122; 12.31%	

*RCD* risk of cardiovascular disease, *SD* standard deviation, *CV* coefficient of variation

Mann-Whitney *U* test. (*P* < 0.05)

Food intake according to food group is shown in Table 4. There was a predominance of low intake of foods in the protein group.

**Table 4:** Level of food intake among leprosy patients, according to food group

Food groups	Intake level			*p*
	Low	Intermediate	High	
	*n* (%)	*n* (%)	*n* (%)	
Meat and eggs	84 (100.0)	0 (0.0)	0 (0.0)	Na
Beef	59 (70.2)	22 (26.2)	3 (3.6)	< 0.0001^a^
Chicken	78 (92.9)	6 (7.1)	0 (0.0)	< 0.0001^b^
Pork	84 (100.0)	0 (0.0)	0 (0.0)	Na
Fish	77 (91.7)	7 (8.3)	0 (0.0)	< 0.0001^b^
Game meat	84 (100.0)	0 (0.0)	0 (0.0)	Na
Ox heart	84 (100.0)	0 (0.0)	0 (0.0)	Na
Bull tongue	84 (100.0)	0 (0.0)	0 (0.0)	Na
Bull liver	84 (100.0)	0 (0.0)	0 (0.0)	Na
Shellfish	83 (98.8)	1 (1.2)	0 (0.0)	< 0.0001^b^
Eggs	69 (82.1)	11 (13.1)	4 (4.8)	< 0.0001^a^
Beef jerky	44 (52.4)	22 (26.2)	18 (21.4)	0.0009^a^
Sausage/Salami	79 (94.0)	2 (2.4)	3 (3.6)	< 0.0001^a^
Sausage	84 (100.0)	0 (0.0)	0 (0.0)	Na
Ham	81 (96.4)	1 (1.2)	2 (2.4)	< 0.0001^a^
Bologna	83 (98.8)	1 (1.2)	0 (0.0)	< 0.0001^b^
Corned beef	83 (98.8)	1 (1.2)	0 (0.0)	< 0.0001^b^
Canned sardines	83 (98.8)	1 (1.2)	0 (0.0)	< 0.0001^b^
Milk and milk products	83 (98.8)	1 (1.2)	0 (0.0)	< 0.0001^b^
Whole milk	21 (25.0)	3 (3.6)	60 (71.4)	< 0.0001^a^
Full fat yoghurt	79 (94.0)	2 (2.4)	3 (3.6)	< 0.0001^a^
Skimmed milk	79 (94.0)	1 (1.2)	4 (4.8)	< 0.0001^a^
Semi-skimmed milk	84 (100.0)	0 (0.0)	0 (0.0)	Na
Low-fat yoghurt	84 (100.0)	0 (0.0)	0 (0.0)	Na
Cottage cheese	83 (98.8)	1 (1.2)	0 (0.0)	< 0.0001^a^
Yellow cheese	78 (92.9)	5 (6.0)	1 (1.2)	< 0.0001^a^
White cheese	83 (98.8)	1 (1.2)	0 (0.0)	< 0.0001^b^
Legumes	83 (98.8)	1 (1.2)	0 (0.0)	< 0.0001^b^
Bean	26 (31.0)	14 (16.7)	44 (52.4)	0.0003^a^
Soy	84 (100.0)	0 (0.0)	0 (0.0)	Na
Chickpea	84 (100.0)	0 (0.0)	0 (0.0)	Na
Pea	83 (98.8)	1 (1.2)	0 (0.0)	< 0.0001^b^
Lentils	84 (100.0)	0 (0.0)	0 (0.0)	Na
Lettuce	68 (81.0)	14 (16.7)	2 (2.4)	< 0.0001^a^
Raw salad	75 (89.3)	8 (9.5)	1 (1.2)	< 0.0001^a^
Cooked salad	68 (81.0)	14 (16.7)	2 (2.4)	< 0.0001^a^
Oils	83 (98.8)	1 (1.2)	0 (0.0)	< 0.0001^b^
Coconut	80 (95.2)	3 (3.6)	1 (1.2)	< 0.0001^a^
Brazil nut	83 (98.8)	1 (1.2)	0 (0.0)	< 0.0001^b^
Cashew nut	84 (100.0)	0 (0.0)	0 (0.0)	Na
Peanut	84 (100.0)	0 (0.0)	0 (0.0)	Na
Walnuts	84 (100.0)	0 (0.0)	0 (0.0)	Na
Fruits	73 (86.9)	11 (13.1)	0 (0.0)	< 0.0001^b^
Avocado	81 (96.4)	3 (3.6)	0 (0.0)	< 0.0001^b^
Muruci	83 (98.8)	1 (1.2)	0 (0.0)	< 0.0001^b^
Açaí	48 (57.1)	19 (22.6)	17 (20.2)	< 0.0001^a^
Fried foods and snacks	83 (98.8)	1 (1.2)	0 (0.0)	< 0.0001^b^
Salted	78 (92.9)	6 (7.1)	0 (0.0)	< 0.0001^b^
Fried preparations	77 (91.7)	6 (7.1)	1 (1.2)	< 0.0001^a^
Pizza	83 (98.8)	1 (1.2)	0 (0.0)	< 0.0001^b^
Sandwich	81 (96.4)	3 (3.6)	0 (0.0)	< 0.0001^b^
Snacks	84 (100.0)	0 (0.0)	0 (0.0)	Na
Ice cream	83 (98.8)	1 (1.2)	0 (0.0)	< 0.0001^b^

*Na* not applicable

^a^Chi-square test

^b^chi-square test with Yates correction

(*P* < 0.05)

Table 5 shows the correlation between the lipid profile of leprosy patients and food intake. A significant correlation (rs = -0.30; *P* = 0.0264) was only observed between the HDL-c fraction in men and dietary intake of fried foods and snacks.

**Table 5:** Correlation between lipid profile and food intake in leprosy patients, by food group

Lipid profile *r* (*P*)	Food intake				
	Meat and eggs	Milk and milk products	Oils	Fruits	Fried foods and snacks
Total cholesterol (mg/dL)	0.07 (0.5006)	0.06 (0.5670)	0.05 (0.6280)	−0.08 (0.4635)	−0.09 (0.4188)
Triglycerides (mg/dL)	0.15 (0.1758)	−0.04 (0.6936)	−0.03 (0.7809)	0.05 (0.6037)	0.12 (0.2871)
LDL-c (mg/dL)	−0.08 (0.4657)	0.02 (0.8539)	0.06 (0.5964)	−0.04 (0.6869)	0.18 (0.1029)
HDL-c (mg/dL), male	0.16 (0.2439)	−0.06 (0.6380)	0.09 (0.5327)	0.06 (0.6752)	−0.30 (0.0264)
HDL-c (mg/dL), female	0.05 (0.7921)	−0.07 (0.7038)	0.001 (0.9631)	0.23 (0.2262)	0.12 (0.5192)

*r* Spearman correlation

(*P* < 0.05)

## Discussion

There was a predominance of male (65.48%) than female with leprosy in the present study, in agreement with most reports about leprosy [2]. However, Montenegro et al. [16] observed a trend towards a higher proportion of females, which can be explained by the fact that women have gained a more active role in the labour market.

The 50–60 year age group was the main group affected by leprosy, in agreement with some literature [17]. Other literature, however, for example, Reibel et al. [18], studying patients with leprosy in Passos, Minas Gerais, Brazil, observed that the age group most affected by the disease was the 20–64 age group, which includes a portion of the economically active population.

In this study, most of the patients had no defined profession or occupation, a finding reflecting the low education level observed. A low education level is a reality in the northern region of Brazil, which, according to the Brazilian Institute of Geography and Statistics [19], has the second highest illiteracy rate in the country. Similar results have been reported by Montenegro et al. [16]. The percentage of low-income patients receiving 248–496 American dollars was high in the present study (63.10%), in agreement with other reports [20].

Lifestyle habits including alcohol consumption, cigarette use, and physical activity were also evaluated. White and Franco-Paredes [21] observed that 35.74% of leprosy patients consume alcoholic beverages and 36.99% smoke. Similar results were obtained in the present study. Messner and Bernhard [22] reported that smoking causes a reduction in HDL-c associated with an increase in LDL-c and triglycerides, enhancing lipid disorders, and that alcohol exerts various effects on lipid levels, including an increase in triglycerides and HDL-c.

Rafferty [23] emphasized that physical activity improves the quality of life of leprosy patients and contributes to the rehabilitation of physical disabilities. Additionally, physical activity can help patients cope better with social and personal problems. Rao and John’s study [20] found that most leprosy patients (61.5%) performed exercise. In contrast, the present study population was found to be sedentary (76.19%).

The analysis of the biochemical results indicates normal levels of total cholesterol in both forms (PB and MB) of the disease, in agreement with other studies [24]. However, Bansal et al. [25] detected low levels of total cholesterol in the two forms of the disease. Other authors have even reported an increase of total cholesterol in patients with leprosy [26]. These disagreeable data probably occurred due to the different populations studied and the different feeding patterns of each of these populations.

Regarding HDL-c, patients of both genders with the PB and MB forms exhibited mean levels within the normal range. These results corroborate the findings of Moschella [27], who observed an increase in HDL-c in PB and MB patients after the end of multidrug therapy, however, the levels were within the normal range. Bansal et al. [25] also found a significant increase in HDL-c levels in patients with MB and PB forms compared to the control group (*P* ≤ 0.05), with the results consistent with those reported by other authors [8]. In contrast, Gupta et al. [7] detected significantly reduced HDL-c levels in patients with the lepromatous form of leprosy compared to the controls.

Normal LDL-c levels were observed in the two forms of leprosy, with no significant differences between them, in agreement with the results of other studies [7, 27]. Hariprasad et al. [28] detected low LDL-c levels in patients with tuberculoid leprosy compared to the control group. Bansal et al. [25] observed a significant reduction in LDL-c levels in patients with the PB and MB forms when compared to the control group.

Triglyceride levels were found to be normal, although lower mean concentrations were observed in the PB form (123.4 mg/dL) compared to the MB form (147.4 mg/dL), but the difference was not significant. Normal triglyceride levels in leprosy patients have also been reported by other authors [24]. In contrast to these findings, Kumar et al. [8] observed a reduction in triglyceride concentrations in patients with the PB and MB forms when compared to the control group and related this finding to a possible overall reduction of lipids in the disease.

Based on the classification of nutritional status according to the BMI, a significant proportion of the sampled patients had excess weight. According to data from the Brazilian Household Budget Survey (2010) on nutritional status, the prevalence of overweight and obesity is high in Brazil, affecting half the adult population [19]. Similar results have been reported in other studies [16, 29]. In contrast, a high degree of malnutrition has been observed in studies conducted in India (BMI < 17 kg/m^2^) [30, 31]. These discordant data occurred due to the different populations studied and the different feeding patterns of each of these populations.

The measure of WC was used in this study as this parameter indicates the main risk factor for CVD and is an independent risk factor for lipid abnormalities [32]. Altered WC was observed in more than half the patients studied (22 women and 22 men), indicating an increased risk for CVD in both genders. Similar observations were made in Striuk et al.’s study [6] involving adults of both genders, in which WC was the anthropometric indicator that best correlated with altered biochemical variables.

The food intake of the Brazilian population has undergone significant changes in recent decades and has been analysed considering different aspects. The results of studies indicate new perspectives of the dietary pattern related to the prevalence of inadequate consumption of macro- and micronutrients as a result of social, economic, cultural, nutritional, and demographic factors, causing food hazards and health problems [19]. However, this study demonstrated that patients had a low intake of all food groups, including dietary sources of lipids.

The food intake results obtained can be attributed in part to factors associated with dietary customs of patients, who reported popular cultural knowledge in relation to diet and leprosy with the ingestion of certain food groups in an attempt to improve the clinical course of the disease. Rao and John [20] observed changes in the dietary habits of patients after leprosy diagnosis. The main changes in food intake reported involved the meat group (pork, duck, fish, sausage, and eggs) since they are “unhealthy foods” that worsen the disease and are responsible for its dissemination. Montenegro et al. [16] reported similar results in terms of the most consumed foods (chicken meat, whole milk, eggs, and beans), low protein intake, and low intake of legumes and vegetables compared to the recommendation of the Food Guide for the Brazilian Population.

## Conclusions

The risk of leprosy patients developing CVD was low after treatment considering the lipid profile. On the other hand, the risk of acquiring CVDs was observed to be high due to patients’ excess weight and increased WC. This study will guide clinicians in the adequate treatment of patients with leprosy in order to avoid adverse cardiovascular events.

## Additional file

Additional file 1: Multilingual abstracts in the six official working languages of the United Nations. (PDF 700 kb)

## Abbreviations

BMI: Body mass index
CVD: Cardiovascular disease
HDL-c: High-density lipoprotein cholesterol
LDL-c: Low-density lipoprotein cholesterol
MB: Multibacillary
PB: Paucibacillary
SRHU: Specialized Referral Health Unit
WC: Waist circumference
WHO: World Health Organization
