# Supplementary material for: Correlation between therapy and lipid profile of leprosy patients: is there a higher risk for developing cardiovascular diseases after treatment?
Source: Infect Dis Poverty. 2017 May 1;6:82. doi: 10.1186/s40249-017-0295-1 (PMC5410692; doi:10.1186/s40249-017-0295-1)
Supplement: Additional file 1: — Multilingual abstracts in the six official working languages of the United Nations. (PDF 700 kb) [file 40249_2017_295_MOESM1_ESM.pdf]

Translation of the abstract into the six official working languages of the United Nations

العلاقة بين العلاج وتحليل الدهون في الدم في مرضى الجذام: هل هناك خطر أكبر للإصابة بأمراض القلب والأوعية الدموية بعد العلاج؟

روزالبا خامسا ج. سيلفا، رافائيل إس دي أروجو، تينارا ل س آرانو، باولو ديوفان دا سيلفا كوستا، خورخي ر سوزا، خواريز أ. س. كواريسما

ملخص

خلفية: أثر الجذام يقلل من نوعية الحياة المرتبطة بالصحة في المرضى المصابين، حيث يتداخل مع عوامل مختلفة مثل التغذية. درست هذه الدراسة الدهون والحالة التغذوية، وخطر الإصابة بأمراض القلب والأوعية الدموية لدى المرضى الذين خضعوا لعلاج الجذام في البرازيل.

الطرق: أربعة وثمانون من المرضى البالغين من كلا الجنسين تتراوح أعمارهم 20-60 عاما وشخص إصابتهم بالجذام قَليلُ العُصَيَّات أو مُتَعَدِّدُ العُصَيَّات وقد تم اختيار بعد خضوعهم للعلاج متعدد الأدوية. تم جمع البيانات التالية: البيانات الاجتماعية والديموغرافية والسريرية. تناول الطعام؛ القياسات البشرية (الوزن، الطول، محيط الخصر)؛ مكونات الدهون (الكوليسترول، البروتين الدهني عالي الكثافة الكوليسترول [C-HDL] ومنخفض الكثافة الكوليسترول [C-LDL]، الدهون الثلاثية).

النتائج: من بين الذين شملتهم الدراسة، كان هناك غلبة للذكور (65.48٪) الذين تتراوح أعمارهم بين 50-60 عاما، المرضى الذين يبلغ دخلهم 248-496 دولار أمريكي (63.10٪)، المرضى الذين أتموا المرحلة الابتدائية (65.48٪)، المرضى غير النشطون (76.19٪)، غير المدخنين (46.43٪)، المرضى غير متعاطي الخمور (69.05٪). وكانت مستويات (الوسط الحسابي ± الانحراف المعياري) من الكوليسترول الكلي 193.8 ± 29.4مجم / ديسيلتر في شكل قَليلُ العُصَيَّات و203.5 ± 41.7مجم / ديسيلتر في شكل مُتَعَدِّدُ العُصَيَّات. وكان متوسط LDL-C 116.9 ± 22.7مجم / ديسيلتر في المرضى الذين يعانون من قَليلُ العُصَيَّات و121 ± 31.3مجم / ديسيلتر في المرضى الذين يعانون مُتَعَدِّدُ العُصَيَّات. كان الوسط الحسابي لمستويات الدهون الثلاثية 123.4 ± 45.2مجم / ديسيلتر في شكل قَليلُ العُصَيَّات و147.4 ± 88.9مجم / ديسيلتر في شكل مُتَعَدِّدُ العُصَيَّات. وأظهر تقييم الحالة التغذوية أن 41.67٪ من المرضى كانوا أغنياء، في حين أن 55.96٪ كانوا زائدو الوزن. وارتبط تناول الطعام بشكل ملحوظ مع HDL-C في المرضى الذكور (P = 0.0264) ومع الدهون الثلاثية في المرضى فوق الوزن المثالي (P = 0.0049).

الخلاصة: خطر الإصابة بالأمراض القلبية الوعائية يزداد في المرضى ذوي الوزن الزائد والذين يعانون من زيادة في محيط الخصر. وهذه الدراسة ترشد الأطباء إلى العلاج المناسب للمرضى الذين يعانون من مرض الجذام من أجل تجنب الإصابة بالأمراض القلبية الوعائية.

Translated from English version into Arabic by Mahmoud Sami, through

TRANSLATORS
WITHOUT BORDERS

麻风患者的血脂与治疗的相关性：治疗后患心血管疾病的风险较高？

Rosalba V. G. Silva, Rafael S. de Araújo, Tinara L. S. Aarão, Paulo Diovanne da Silva Costa, Jorge R. Sousa, Juarez A. S. Quaresma

摘要

引言：麻风从不同方面影响患者，比如营养，这降低了患者与健康相关的生活质量。本研究调查了在巴西接受麻风治疗的患者，研究其血脂、营养状况和罹患心脑血管病（CVD）的风险。

方法：选择 84 名经多种药物治疗的少菌型(PB)麻风和多菌型(MB)麻风的成年患者，男女均有，年龄在 20 到 60 岁之间。收集以下数据：社会人口统计学和临床数据、食物摄入量、体检数据（体重、身高、腰围）、血脂成分（总胆固醇、高密度脂蛋白胆固醇、低密度脂蛋白胆固醇、甘油三酯）。
结果：在研究人群中，50-60 岁的男性患者占 65.48%，收入为 248-496 美元的占 63.10%，小学毕业的患者占 65.48%，非活动性的患者占 76.19%，不吸烟的占 46.43%，非饮酒者的占 69.05%。少菌型和多菌型麻风患者的总胆固醇水平分别为 193.8±29.4 mg/dL 和 203.5±41.7 mg/dL，低密度脂蛋白胆固醇水平分别为 116.9 ± 22.7mg/dL、121 ± 31.3mg/dL，甘油三酯水平分别为 123.4 ± 45.2mg/dL、147.4 ± 88.9mg/dL。营养状况的评估结果显示，41.67%的患者是富营养的,而 55.96%的患者超重。在男性患者中，食物摄入量与患者高密度脂蛋白胆固醇水平(P = 0.0264)和超重患者的甘油三酯显著相关(P = 0.0049)。
结论：体重和腰围增加均会增加罹患心血管病的风险，本研究将指导临床医生对麻风患者进行充分治疗，以避免不良心血管疾病的发生。

Translated from English version into Chinese by Lei Sun, edited by Pin Yang

CHINESE CENTER FOR DISEASE CONTROL AND PREVENTION
NATIONAL INSTITUTE OF PARASITIC DISEASES

Corrélation entre le traitement et le profil lipidique des patients atteints de la lèpre: Le risque de développer des maladies cardiovasculaires est-il plus élevé après le traitement?

Rosalba V. G. Silva, Rafael S. de Araújo, Tinara L. S. Aarão, Paulo Diovanne da Silva Costa, Jorge R. Sousa, Juarez A. S. Quaresma

RÉSUME
Contexte: La lèpre a un impact considérable sur la qualité de vie liée à la santé des patients, et perturbe de nombreux domaines de leur vie, tel que la nutrition par exemple. Dans le cadre de cette étude, les auteurs ont examiné le profil lipidique, le statut nutritionnel, ainsi que le risque accru de maladies cardiovasculaires (MCV) chez les patients ayant reçu un traitement contre la lèpre au Brésil.
Méthode: Les auteurs ont sélectionné quatre-vingt-quatre patients adultes, des deux sexes, âgés entre 20 et 60 ans, et chez qui on a diagnostiqué la lèpre paucibacillaire (PB) et multibacillaire (MB), après qu’ils aient reçu un traitement par polychimiotherapie (PCT). Pour les besoins de l’étude, les auteurs ont recueilli des renseignements concernant des données cliniques et sociodémographiques, l’apport nutritionnel et les mesures anthropométriques (poid, taille, et tour de taille), ainsi que les composants du profil lipidique (cholestérolémie totale, cholestérol à lipoprotéine de haute densité (HDL), cholestérol à lipoprotéine de faible densité (LDL), ainsi que les triglycérides).
Résultats: La population étudiée était majoritairement masculine. Les patients âgés entre 50 et 60 ans représentaient 65,48% de la population étudiée. 63,10% d’entre eux percevaient un salaire moyen de 248-496 dollars; 65,48 % avaient arrêté leurs études à l’école élémentaire, tandis que 76,19% étaient inactifs. La proportion des patients non-fumeur s’élevait 46,43% ; 69,05% des d’entre eux ne consommaient pas d’alcool. Le niveau total de cholestérolémie (moyenne ± écart-type) 193,8 ± 29.4mg/dL chez les patients atteints de la lèpre PB, et de 203,5 ± 41.7mg/dL chez les

patients atteints de la lèpre MB. La moyenne de la LDL-c s'élevait à 116.9 ± 22.7mg/dL chez les patients atteints de la lèpre PB et à 121 ± 31.3mg/dL chez les patients atteints de la lèpre MB. La moyenne du taux de triglycéride s'élevait à 123.4 ± 45.2mg/dL chez les patients atteints de lèpre PB contre 147.4 ± 88.9mg/dL chez les patients atteints de lèpre MB. L'évaluation du statut nutritionnel a démontré que 41,67% des patients étaient eutrophiques, tandis que 55,96% d'entre eux était en surpoids. Les auteurs ont observé une forte corrélation entre l'alimentation, le cholestérol à HDL chez les patients de sexe masculin (P = 0.0264) et les triglycérides chez les patients dont le poids est supérieur au poids idéal recommandé (P = 0.0049).

Conclusion: Selon les auteurs de cette étude, les patients en surpoids et dont le tour de taille est élevé étaient plus à risque de contracter une MCV. L'objectif de cette étude est de servir de guide aux cliniciens et de leur permettre de fournir un traitement adéquat aux patients atteints de lèpre, et ce afin de prévenir toute complication cardiovasculaire indésirable.

Translated from English version into French by Patricia Roth, through

TRANSLATORS WITHOUT BORDERS

Связь между терапией и липидным профилем больных проказой: существует ли повышенный риск развития сердечно-сосудистых заболеваний после лечения?

Розальба В. Г. Силва (Rosalba V. G. Silva), Рафаэль С. де Араухо (Rafael S. de Araújo), Тинара Л. С. Аарао (Tinara L. S. Aarão), Пауло Диованне де Силва Коста (Paulo Diovanne da Silva Costa), Хорхе Р. Суса (Jorge R. Sousa), Хуарес А. С. Куарежма (Juarez A. S. Quaresma)

Аннотация

Обоснование исследования. Проказа ухудшает связанное с состоянием здоровья качество жизни больных, затрагивая разные факторы, такие как питание. В данном исследовании изучались липидный профиль, пищевой статус и риск развития сердечно-сосудистого заболевания (ССЗ) у больных, получивших лечение от проказы в Бразилии.

Методы. Были отобраны восемьдесят четыре взрослых пациента обоих полов в возрасте от 20 до 60 лет с диагнозом олигобациллярная (ОБ) или мультибациллярная (МБ) проказа, которые получили комбинированную лекарственную терапию. Была собрана следующая информация: социально-демографические и медицинские данные, приём пищи, антропометрические показатели (вес, рост и окружность талии), составляющие липидного профиля (общий холестерин, липопротеиды высокой плотности (ЛПВП), липопротеиды низкой плотности (ЛПНП) и триглицериды).

Результаты. Среди отобранных больных преобладали мужчины (65,48%) от 50 до 60 лет; больные с доходом 248 – 496 долларов США (63,10%); больные, закончившие начальную школу (65,48%), бездействующие больные (76,19%), некурящие (46,43%) и непьющие (69,05%) больные. Уровни (средняя величина ± стандартное отклонение) общего холестерина составили 193,8 ± 29,4 мг/дл для больных с ОБ и 203,5 ± 41,7 мг/дл для больных

с МБ. Средняя величина ЛПНП была 116,9 ± 22,7 мг/дл для больных с ОБ и 121 ± 31,3 мг/дл для больных с МБ. Средние уровни триглицеридов были 123,4 ± 45,2 мг/дл для больных с ОБ и 147,4 ± 88,9 мг/дл для больных с МБ. Оценка пищевого статуса показала, что состояние 41,67% больных было нормальным и 55,96% пациентов имели избыточный вес. Приём пищи был в значительной степени связан с ЛПВП у больных мужского пола (P = 0,0264) и с триглицеридами у больных, вес которых превышал идеальный (P = 0,0049).
Заключение. Наблюдался высокий риск развития ССЗ вследствие избыточного веса больных и увеличенной окружности талии. Данное исследование послужит руководством для врачей-клиницистов в отношении надлежащего лечения больных проказой во избежание нежелательных явлений для сердечно-сосудистой системы.

Translated from English version into Russian by Elena Alieva, through

Correlación entre terapia y perfil lipídico de pacientes de lepra: ¿existe un mayor riesgo de desarrollo de enfermedades cardiovasculares tras el tratamiento?

Rosalba V. G. Silva, Rafael S. de Araújo, Tinara L. S. Aarão, Paulo Diovanne da Silva Costa, Jorge R. Sousa, Juárez A. S. Quaresma

RESUMEN

Información de referencia: La lepra reduce la calidad de vida de los pacientes afectados e interfiere con diferentes factores, como la nutrición. En este estudio se investigó el perfil lipídico, el estado nutricional y el riesgo de enfermedad cardiovascular en pacientes sometidos a tratamiento para la lepra en Brasil.
Métodos: Se seleccionó a 84 pacientes adultos de ambos sexos, con edades de 20 a 60 años y diagnosticados de lepra paucibacilar (PB) o multibacilar (MB) tras haber recibido un tratamiento con diversos fármacos. Se recogieron los siguientes datos: datos sociodemográficos y clínicos; ingestión de alimentos; medidas antropométricas (peso, altura y perímetro de la cintura); y componentes del perfil lipídico (colesterol total, colesterol de lipoproteína de alta densidad (HDL-c), colesterol de lipoproteína de baja densidad (LDL-c) y triglicéridos).
Resultados: Entre la población estudiada hubo una predominancia de varones (65,48%) de 50 a 60 años, pacientes con unos ingresos de 248–496 dólares estadounidenses (63,10%), pacientes que habían finalizado sus estudios primarios (65,48%), pacientes inactivos (76,19%), no fumadores (46,43%) y pacientes que no beben alcohol (69,05%). Los niveles (medio ± desviación estándar) del colesterol total fueron de 193,8 ± 29,4mg/dL para el tipo PB y 203,5 ± 41,7mg/dL para el tipo MB. El LDL-c medio fue 116,9 ± 22,7mg/dL en pacientes de PB y 121 ± 31,3mg/dL en pacientes de MB. Los niveles medios de triglicéridos fueron 123,4 ± 45,2mg/dL para el tipo PB y 147,4 ± 88,9mg/dL para el tipo MB. La evaluación del estado nutricional indicó que el 41,67% de los pacientes era eutrófico, mientras que el 55,96% tenía exceso de peso. La ingestión de alimentos se

relacionó de manera significativa al HDL-c en los pacientes varones (*P* = 0,0264) y con los triglicéridos en pacientes con un peso superior al ideal (*P* = 0,0049).
Conclusión: Se observe que el riesgo de contraer enfermedades cardiovasculares era mayor debido al exceso de peso y al mayor perímetro de la cintura de los pacientes. Este estudio servirá de guía para los médicos para el tratamiento adecuado de pacientes con lepra con el fin de evitar episodios adversos de tipo cardiovascular.

Translated from English version into Spanish by Sergio Lorenzi, through

TRANSLATORS
WITHOUT BORDERS
